# Serum-derived exosomal microRNAs as biomarkers for postoperative delirium

**DOI:** 10.3389/fnins.2025.1525230

**Published:** 2025-02-28

**Authors:** Maokai Xu, Yingjie Chen, Yujun Lin, Danfeng Wang, Xiaochun Zheng

**Affiliations:** ^1^Department of Anesthesiology, Fujian Provincial Hospital, Fuzhou University Affiliated Provincial Hospital, Fuzhou, China; ^2^Department of Critical Care Medicine, Fuzhou University Affiliated Provincial Hospital, Fuzhou, China

**Keywords:** postoperative delirium, cognitive decline, biomarkers, microRNA, serum exosomes

## Abstract

**Introduction:**

Postoperative delirium (POD) is a frequent and challenging complication in elderly surgical patients, marked by abrupt cognitive and attentional disturbances. Current POD diagnosis depends on clinical assessments that are time-intensive and lack predictive accuracy before surgery. Although previous research has explored biomarkers such as neuroinflammatory factors and Alzheimer’s-related proteins to enhance POD prediction, single molecular markers have proven insufficient for reliable prognosis.

**Methods:**

This study investigated serum exosomal miRNA expression profiles in postoperative patients to assess their association with POD. We compared miRNA expression between POD and non-POD groups through cognitive assessments and serum analyses. Additionally, enrichment analysis was conducted to determine the biological pathways regulated by differentially expressed miRNAs.

**Results:**

Our analysis identified 57 miRNAs with significantly altered expression between POD and non-POD patients, including 16 upregulated and 41 downregulated miRNAs in the POD group. Enrichment analysis revealed that these miRNAs are involved in genes regulating neurotrophin signaling, neuroactive ligand-receptor interactions, and pathways that influence neuronal plasticity and cell viability.

**Discussion:**

This study highlights specific miRNAs as potential biomarkers for POD and suggests their involvement in the underlying mechanisms of cognitive decline following surgery. By enhancing diagnostic capabilities and identifying potential therapeutic targets, our findings could lead to more effective POD management strategies for elderly patients. Further research is recommended to validate these miRNAs and evaluate their clinical utility for predictive screening and therapeutic interventions.

## 1 Introduction

Postoperative delirium (POD) is an acute, fluctuating disturbance in attention and cognition that typically develops within days after surgery, increasing the risks of prolonged hospital stays and, in severe cases, long-term cognitive impairment. The causes of POD are multifaceted, often involving inflammation, metabolic disturbances, and age-related vulnerability, with incidence rates reaching up to 50% in patients aged 65 and older (Dobson, 2020). As the World Health Organization predicts that 22% of the global population will be aged 60 and above by 2050 (Padeiro et al., 2022), the number of elderly surgical patients is expected to rise, likely leading to an increased incidence of POD and placing significant strain on healthcare systems.

At present, the diagnosis of POD is primarily dependent on patient-reported symptoms, mental status evaluations, and clinical assessments. These methods can be time-intensive and are limited by the absence of preoperative diagnostic techniques. Currently, a variety of biomolecules and pathological markers have been identified as POD predictors, with many linked to neuroinflammation, cognitive decline, and proteins associated with Alzheimer’s disease (AD) (Androsova et al., 2015a; Nation et al., 2019). For example, research suggests that older males possessing the APOE4 allele may exhibit a higher susceptibility to cognitive decline following surgery compared to females with the same genetic variant (Androsova et al., 2015a; Schaefer et al., 2019; Dunne et al., 2021). Additional biomarkers, including S100β and neuron-specific enolase (NSE), have been linked to POD, although the underlying mechanisms and genetic determinants remain poorly understood. Nonetheless, the reliance on a singular molecular indicator proves inadequate for predicting the intricate nature of this condition, underscoring the necessity for continued research aimed at developing more rapid and reliable diagnostic methodologies.

Non-coding microRNAs (miRNAs), which are small RNA molecules comprising 20–25 nucleotides, serve critical regulatory functions in numerous biological processes by binding to target messenger RNAs (mRNAs) and modulating their stability and translation into proteins (Saliminejad et al., 2019). Importantly, miRNAs possess the capability to regulate multiple genes at distant loci, thereby emphasizing their broad functional impact (Theil et al., 2019). Recent studies have elucidated the involvement of miRNAs in a variety of health conditions, such as metabolic disorders and infectious diseases. A growing body of evidence associates miRNAs with neurodegenerative diseases, including AD, Parkinson’s disease (PD), and amyotrophic lateral sclerosis (Silvestro et al., 2019; Swarbrick et al., 2019; Salemi et al., 2023). Specific miRNAs, such as miR-107, miR-9, and miR-125b, have been identified in blood, cerebrospinal fluid (CSF), and brain tissues, and have been proposed as potential biomarkers for AD (Silvestro et al., 2019). In the context of PD, more than 15 miRNAs exhibit dysregulation across the brain, CSF, and blood, further underscoring their potential utility as diagnostic markers for neurodegenerative disorders (Salemi et al., 2023). Peripheral surgical trauma, in conjunction with anesthesia, contributes to the onset of POD, suggesting that circulating molecules such as small RNAs may have distal regulatory functions (Gao et al., 2020). Consequently, exosomal miRNAs detected in the bloodstream could serve as novel, minimally invasive biomarkers for patients undergoing major surgical procedures.

This study sought to elucidate the differential expression profiles of serum exosomal miRNAs in patients with POD relative to those without POD following surgical procedures. By conducting a clinical evaluation of 50 postoperative patients, we analyzed data derived from cognitive assessments, including the Mini-Mental State Examination (MMSE), the Stroop Color and Word Test (SCWT), and the Trail Making Test (TMT). Consequently, we identified 57 distinct serum exosomal miRNAs in POD patients that demonstrated significant alterations in expression compared to non-POD patients. Among these 57 miRNAs, 16 showed significantly elevated expression, while 41 demonstrated decreased expression. Kyoto Encyclopedia of Genes and Genomes (KEGG) enrichment and Gene Ontology (GO) analysis have revealed that the genes regulated by these 57 miRNAs are involved in the neurotrophin signaling pathway, neuroactive ligand-receptor interaction, and other pathways related to cell viability and neuronal plasticity. In conclusion, we have identified distinct alterations in serum exosomal miRNAs in patients who develop POD following surgery. These specific alterations could serve as potential preoperative biomarkers for POD, while the aberrantly expressed miRNAs may offer promising therapeutic targets.

## 2 Method

### 2.1 Patients

The study was approved by the Medical Ethics Committee of Fuzhou University Affiliated Provincial Hospital, and written informed consent was obtained from each participant before enrollment. Participants aged 60–80 included 50 patients who had undergone total hip arthroplasty or total knee arthroplasty. All surgeries were performed at Fuzhou University Affiliated Provincial Hospital. Exclusion criteria were pre-existing neurological disease [Mini-Mental State Examination (MMSE) score < 24], a history of antidepressant or anti-anxiety medication use, anticipated challenges with neuropsychological assessment (such as severe visual or hearing impairment), deep hypothermic circulatory arrest during surgery, perioperative insulin therapy, and reduced ventricular function (ejection fraction <30%).

### 2.2 Anesthesia protocol and surgery types

All patients received standard perioperative care, and all procedures were conducted by the same surgical team at Fuzhou University Affiliated Provincial Hospital. The anesthesia protocol followed a standard pattern, using midazolam (0.05–0.1 mg/kg), fentanyl (5–8 g/kg), and etomidate (0.2–0.3 mg/kg) for induction. Fentanyl (0.1–0.15 g/kg/min), midazolam (0.6–1 g/kg/min), sevoflurane (0.5–1%) in oxygen, and vecuronium (0.05 mg/kg every 30 min) were used for maintenance anesthesia. All patients underwent standard monitoring, including electrocardiography, pulse oximetry, end-tidal carbon dioxide, nasopharyngeal temperature, central venous and arterial blood pressure, arterial blood gasses, cardiac output, and bispectral index. After surgery, all patients were transferred for close monitoring and postoperative care.

### 2.3 Neuropsychological assessment

Patients were first screened using the MMSE to exclude those with severe cognitive impairment. Eligible participants then underwent neuropsychological testing 1 day before and 1 day after surgery. Based on validated Chinese standardized tests, the assessments evaluated attention, motor skills, executive function, learning, and memory (Fiorillo et al., 2020; Ni et al., 2022). The SCWT was used to evaluate attention and distraction resistance by recording time and errors, while the TMT assessed mental processing speed and visual scanning through completion times. A decline in postoperative scores of at least 20% compared with preoperative scores was defined as POD in this study.

### 2.4 Patient information and sample preparation

The study collected demographic and clinical data, including age, weight, sex, body mass index (BMI), and ejection fraction (EF). The study included 10 patients from Fuzhou University Affiliated Provincial Hospital, comprising three healthy individuals and seven patients who developed POD, and the patient characteristics were summarized in Table 1. Clinical approval was obtained from the hospital’s ethics committee. Blood samples (10 mL) were collected 1 day before surgery and 1 day afterward (at 08:00 a.m. after overnight fasting). Samples were placed in anticoagulant tubes, centrifuged at 4°C, first at 300 × *g* for 5 min, then at 1,200 × *g* for 20 min. Plasma was stored at −80°C for further analysis.

**Table 1:** Characteristics of the patients.

Characteristics	Non-POD group (*n* = 3)	POD group (*n* = 7)	*P*-value
Age, years	67.33 ± 3.22	65.43 ± 4.39	0.52
Female, *n*	1/3	2/7	0.88
Height, cm	170.3 ± 9.018	167.3 ± 10.59	0.68
Weight, kg	69.67 ± 5.859	68.71 ± 8.440	0.87
BMl, kg/cm^2^	24.19 ± 3.80	24.61 ± 2.69	0.84
EF, %	58.33 ± 5.13	57.86 ± 6.77	0.92
NYHA			
I	2	5	
II	1	2	
Surgical time, min	124.3 ± 28.02	130.6 ± 30.12	0.77

POD, postoperative cognitive dysfunction; BMI, body mass index; EF, ejection fraction; NYHA, New York Heart Association.

### 2.5 Exosome isolation

To isolate exosomes from plasma, samples were centrifuged at 500 × *g* for 5 min at 4°C, followed by transfer to a new tube and centrifugation at 2,000 × *g* for 10 min at 4°C. The supernatant was collected and centrifuged at 10,000 × *g* for 30 min at 4°C to remove shed microvesicles (200–1,000 nm). The supernatants were filtered through a 0.22 μm membrane filter (EMD Millipore) and centrifuged at 1,00,000 × *g* for 2 h at 4°C. The exosome pellet was washed once with 1× PBS, centrifuged again at 1,00,000 × *g* for 2 h, resuspended in 1× PBS, and stored at −80°C for future use.

### 2.6 Exosome characterization

Exosomes isolated from plasma were washed with PBS, filtered through a 0.22 μm membrane, and ultracentrifuged at 1,00,000 × *g* for 2 h at 4°C. The pellet was resuspended in PBS with 2% glutaraldehyde for 5 min at 4°C, placed on carbon-coated electron microscopy grids, contrasted with 0.5% uranyl acetate, and examined using a Tecnai G2 Spirit 120 kV electron microscope. Exosome characterization involved transmission electron microscopy (TEM) to observe morphology, nanoparticle tracking analysis (NTA) to determine size and concentration, and Western blotting (WB) to confirm the presence of the exosome marker CD81 (AF2428, Beyotime, China).

### 2.7 MiRNA sequencing and analysis

Raw data were generated after sequencing, image analysis, base calling and quality filtering on sequencer. Initial quality control was conducted using Q30, followed by trimming of adaptor sequences with Cutadapt software (v1.9.3), retaining reads of at least 15 nucleotides (Martin, 2011). A maximum of one mismatch was detected when the trimmed reads were aligned to the merged pre-miRNA databases (known pre-miRNAs from miRBase plus the newly predicted pre-miRNAs) using mirdeep software (Mackowiak, 2011). The raw expression levels of mature miRNAs were determined by the number of mapped tags, and read counts were normalized using the TPM (tags per million aligned miRNAs) method. To predict novel miRNAs, trimmed reads from all samples were pooled and miRDeep2 software (v2.0.0.5) was used (Mackowiak, 2011). Fold change and *p*-value were used to filter the differentially expressed miRNA between two groups. The miRNA target prediction software, cytoscape (v2.8.0), was used to plot the miRNA-target networks (Smoot et al., 2011), and the GO and KEGG pathway analysis were performed based on the differentially expressed miRNA target genes.

### 2.8 MiRNA isolation and sequencing

The miRNA sequencing was conducted by CloudSeq Inc. (Shanghai, China). Small RNA libraries were prepared using the GenSeq^®^ Small RNA Library Prep Kit (GenSeq, Inc.), following the manufacturer’s protocol. In short, 3′ and 5′ adaptors were sequentially attached to RNA samples, which were then reverse-transcribed into cDNA and amplified by PCR. Following amplification, the cDNA libraries were size-selected for miRNA fragments prior to sequencing, which was subsequently performed on the designated platform.

### 2.9 GO and KEGG pathway analysis

To explore the biological functions and pathways associated with differentially expressed miRNAs, GO and KEGG pathway enrichment analyses were performed. Target genes for the miRNAs were predicted using established databases (TargetScan, miRDB, and miRTarBase) based on sequence matching and validation evidence. GO analysis categorized these target genes into biological process (BP), cellular component (CC), and molecular function (MF) terms, with significance determined by a Benjamini–Hochberg adjusted *p*-value <0.05. KEGG pathway analysis was then conducted to identify relevant pathways using DAVID, with significant pathways identified at an adjusted *p*-value threshold of <0.05. Enriched GO terms and KEGG pathways were visualized to illustrate key biological processes and pathways affected by the miRNAs, providing insights into their roles in cognitive dysfunction mechanisms.

### 2.10 Statistical analysis

For statistical analysis, differentially expressed miRNAs were identified using DESeq2, a tool specifically designed for RNA-seq count data. It employs a negative binomial generalized linear model to address the discrete and overdispersed nature of RNA-seq data, with normalization using the median-of-ratios method to account for sequencing depth and sample variability. Significance was determined by setting a false discovery rate (FDR) threshold of <0.05, adjusted using the Benjamini-Hochberg method to control for multiple testing. To evaluate functional enrichment, we performed GO and KEGG pathway analyses using hypergeometric tests, assessing the overrepresentation of specific pathways or terms among the targeted genes of differently expressed miRNAs compared to the background set of all detected miRNAs, with a significance threshold of *p* < 0.05. Principal component analysis (PCA) was performed to visualize expression patterns and assess overall sample variation. Before PCA, the data were log-transformed and mean-centered to ensure compatibility with the assumptions of PCA. Statistical analyses were conducted using R (version 4.4.1) or Python (version 3.12), with results presented as mean ± standard deviation where applicable.

## 3 Results

### 3.1 Patients included and perioperative characteristics of patients

**Figure 1:**
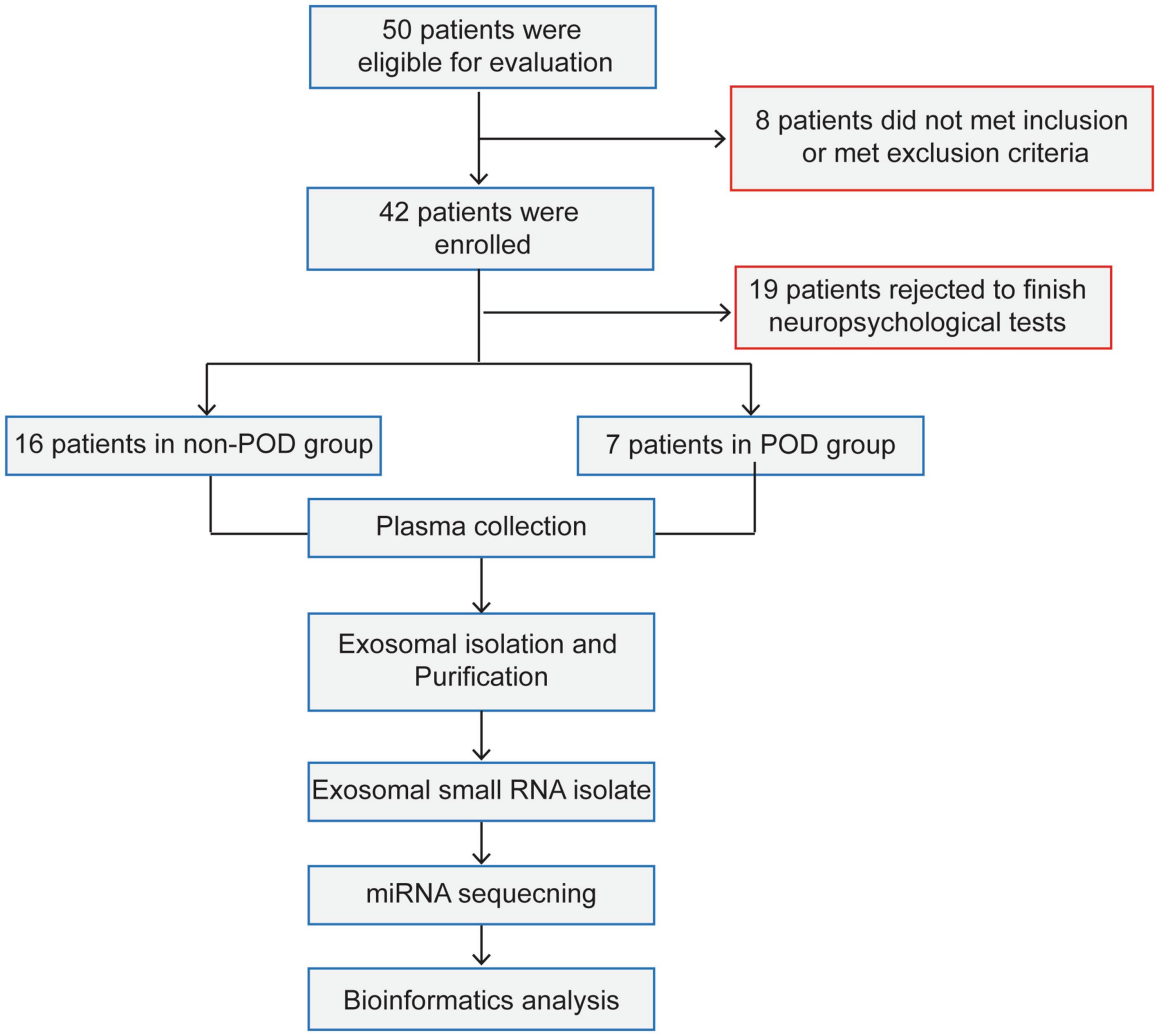
The process flow diagram of experiment. POD, postoperative delirium. Non-POD, the patients without postoperative delirium.

The study involved 50 patients and only 23 completed the cognitive assessments. The remaining 27 patients did not complete the neuropsychological assessments for the following reasons: 8 patients either did not meet the inclusion criteria or were excluded based on the exclusion criteria, while 19 patients declined to participate in the neuropsychological tests. The reasons for declining participation were primarily related to personal preference or concerns regarding the time commitment required for the assessments (Figure 1). As a result, 7 patients (30.4%) exhibited POD 1 day after surgical intervention and anesthesia. Participants were categorized into two distinct groups according to their cognitive test outcomes: the POD group (*n* = 7) and the non-POD group (*n* = 16). The study methodology was depicted in Figure 1, while the perioperative characteristics of the patient cohort were detailed in Table 1. Statistical analysis revealed no significant differences between the groups concerning age, sex, height, weight, BMI, EF, New York Heart Association (NYHA) classification of heart failure, or duration of surgery (*p* > 0.05). For further isolation of plasma exosomes and sequencing of serum exosomal miRNAs, 3 patients from the non-POD group and 7 patients from the POD group were selected.

### 3.2 Exosome characterization

**Figure 2:**
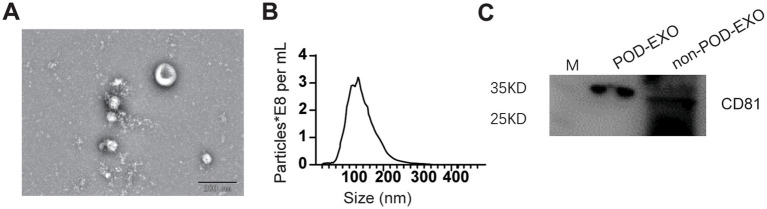
Confirmation of serum exosomes via ETM, NTA, and WB. **(A)** Representative ETM image of exosomes from POD patients. Scale bar: 200 nm. **(B)** NTA analysis of exosomes from POD patients. **(C)** Representative WB image showing CD81.

To investigate miRNAs in serum exosomes, the serum exosomes were isolated using the methods described in “Method section 2.8.” The isolated plasma exosomes were characterized utilizing TEM, NTA, and WB. As illustrated in Figure 2, TEM analysis demonstrated that the exosomes derived from patients with POD exhibited a diameter of approximately 110 nm, whereas NTA measurements indicated an average size of approximately 120 nm. WB analysis further verified the presence of the exosomal marker CD81 in both POD and non-POD patient samples.

### 3.3 Profiling of miRNAs in the plasma exosomes of patients

RNA concentration and purity were assessed using a Nanodrop ND-1000 spectrophotometer. Samples with an A260/A280 and A260/A230 absorbance ratio greater than 1.8 and a total RNA quantity of at least 10 μg were deemed suitable for further analysis (Supplementary Table 1). Among the samples, seven were from patients with POD, and three were selected from 16 non-POD patients. These samples were subsequently used for miRNA expression profiling and comparative analysis.

**Figure 3:**
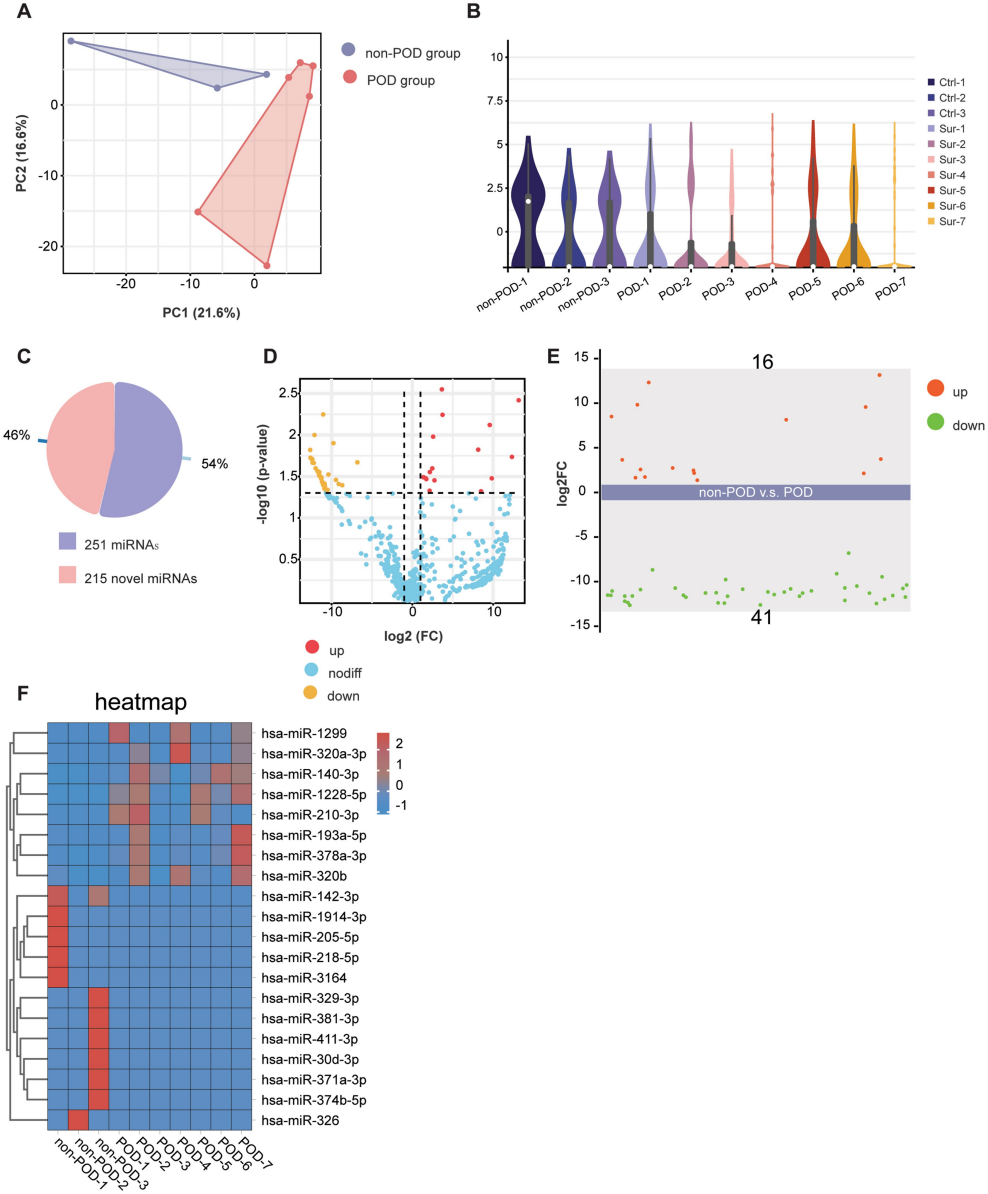
Profiling of serum exosomal miRNAs in POD and non-POD patients. **(A)** PCA results showing the distribution of serum exosomal miRNAs. Each color represents a different group: violet for POD patients (*n* = 7) and red for non-POD patients (*n* = 3). **(B)** Violin plot displaying the distribution of serum exosomal miRNAs across different groups. **(C)** The pie chart displays the proportion of known and novel miRNAs identified in the study. **(D)** The volcano plot illustrates the relationship between the magnitude of change (log2 fold change) and statistical significance (−log10 *p*-value) for the detected miRNAs. **(E)** This scatter plot displays the expression levels of miRNAs between the POD group and the non-POD group. A total of 57 miRNAs were analyzed, with 16 miRNAs significantly upregulated and 41 miRNAs significantly downregulated in the POD group compared to the non-POD group. **(F)** This heatmap illustrates the expression levels of differentially expressed miRNAs in the POD and non-POD groups.

We used PCA to evaluate the overall relationships between samples. The PCA plot reveals notable differences between the POD and non-POD groups along the PC1 and PC2 axes (Figure 3A). The first two principal components, PC1 and PC2, explain 21.6 and 16.6% of the total variance, respectively (Figure 3A). The variance (21.6%) explained by PC1 suggests that this component captures the largest variation in the data, likely reflecting the primary differences between the two groups. Similarly, PC2, which explains an additional 16.6% of the variance, provides further differentiation, highlighting secondary factors that may contribute to the observed distinctions between the groups. These findings suggest that the POD and non-POD groups exhibit notable differences in miRNA expression patterns, with the first two components capturing the majority of this variability. We further used violin plots to illustrate the expression distribution of detected miRNAs across different groups. The violin plots reveal distinct patterns of miRNA expression between the POD and non-POD groups. Specifically, compared to the non-POD group, the miRNA expression in the POD group was more concentrated, with narrower distribution ranges and fewer extreme values (Figure 3B). This suggests that the miRNA expression profile in the POD group is more homogenous, indicating a potential biomarker pattern associated with postoperative cognitive dysfunction. This difference suggests that the categories of miRNA expression are likely to change after patients develop POD following anesthesia and surgery.

For the miRNA sequencing results, we identified a total of 466 distinct miRNAs, comprising 251 known miRNAs and 215 novel miRNAs (Figure 3C). A volcano plot was generated to visualize the significant differences between the POD and non-POD groups, using a Logarithm fold change threshold of ≥2.0 (Log2FC > 1) and a *p*-value threshold of <0.05 (Figure 3D). Among the 251 known miRNAs, 57 showed statistically significant changes (*p* < 0.05 and log2|FC| > 1), including 16 miRNAs that were significantly upregulated and 41 that were significantly downregulated (Figures 3D,E). Corresponding statistical details for these miRNAs, including *p*-values and log2FC values, are presented in Supplementary Table 2 to provide additional clarity and transparency. Additionally, we selected the top 8 upregulated miRNAs, including has-miR-1299, has-miR-320a-3p, has-miR-140-3p, has-miR-1228-5p, has-miR-210-3p, has-miR-193a-5p, has-miR-378a-3p, has-miR-320b, and the top 12 downregulated miRNAs, including has-miR-142-3p, has-miR-1914-3p, has-miR-205-5p, has-miR-218-5p, has-miR-3164, has-miR-329-3p, has-miR-381-3p, has-miR-411-3p, has-miR-30d-3p, has-miR-371a-3p, has-miR-374b-5p, and has-miR-326 for visualization of differential expression through a heatmap, as shown in Figure 3F. The heatmap highlights distinct expression patterns, with upregulated miRNAs predominantly clustering in the POD group and downregulated miRNAs showing lower expression levels compared to the non-POD group. This clear segregation between the two groups underscores the differential regulation of these miRNAs in the context of POD. In summary, these results clearly illustrated the changes in miRNA expression patterns in the POD group, suggesting that these miRNAs may play a significant role in the regulation of postoperative cognitive dysfunction.

### 3.4 Target gene enrichment and pathway analysis of detected miRNAs

**Figure 4:**
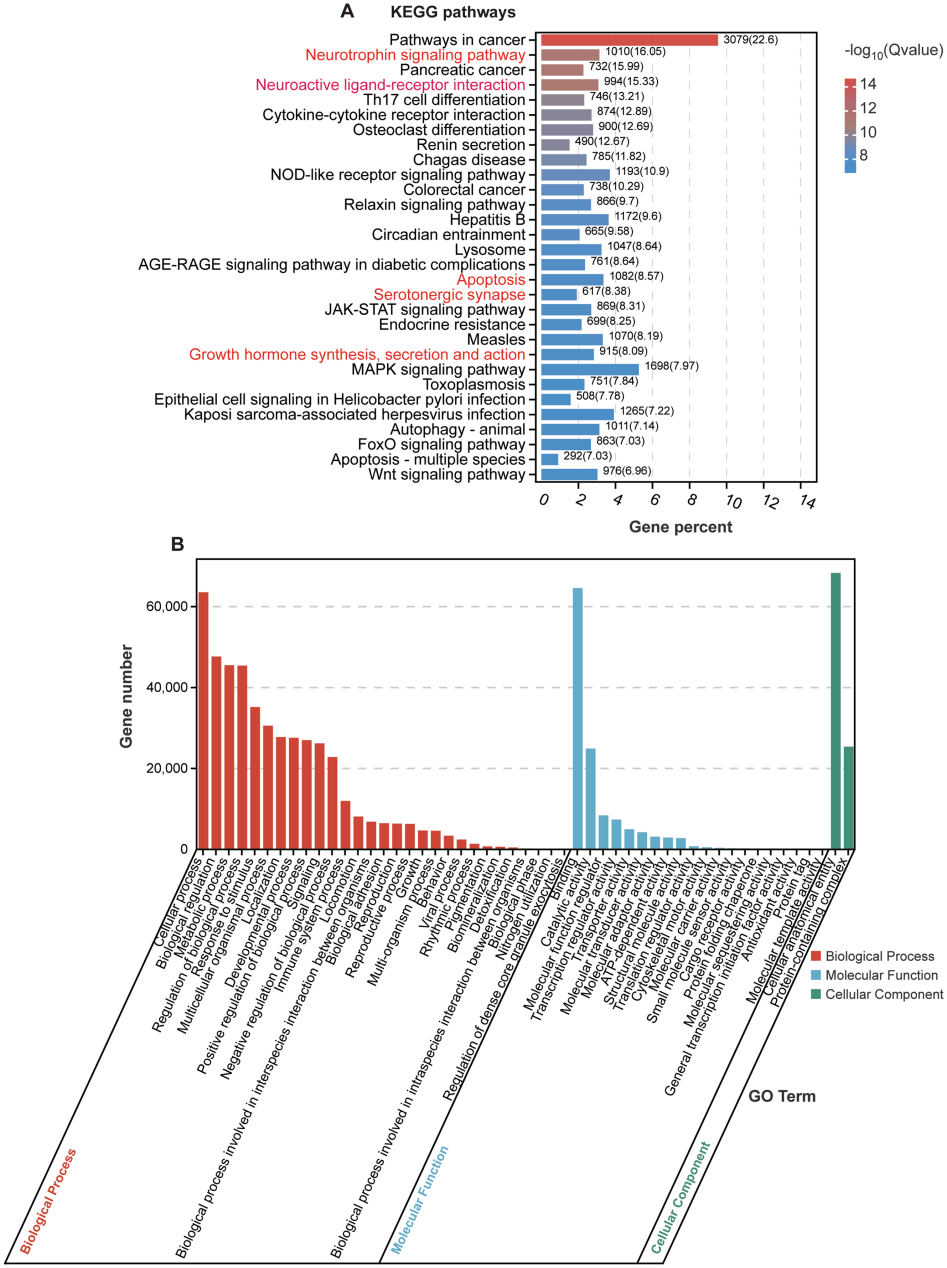
KEGG and GO analyses of enriched signaling pathways. **(A)** KEGG pathway analysis of enriched genes regulated by significantly expressed miRNAs. **(B)** GO analysis of target genes regulated by significantly expressed miRNAs.

We analyzed the KEGG and GO pathways for the 57 detected miRNAs, focusing on their roles in various biological processes and disease mechanisms. Through KEGG pathway enrichment analysis, we identified two significantly enriched signaling pathways: the “Neurotrophin signaling pathway,” which is vital for neuron survival, growth, differentiation, and synaptic plasticity through neurotrophins such as NGF, BDNF, NT-3, and NT-4 (Reichardt, 2006), and the “Neuroactive ligand-receptor interaction” pathway, which mediates the interaction between neuroactive ligands (including neurotransmitters and hormones) and their specific receptors, facilitating synaptic transmission, neuronal communication, and modulation of brain activity (Figure 4A) (Neff et al., 2021a).

Notably, several significantly altered miRNAs identified in our analysis are closely associated with these pathways. For example, has-miR-1299 and has-miR-193a-5p, both significantly upregulated, are predicted to regulate genes involved in the neurotrophin signaling pathway, which could affect synaptic plasticity and neuronal survival. On the other hand, has-miR-142-3p and has-miR-218-5p, which were significantly downregulated, are linked to the neuroactive ligand-receptor interaction pathway, potentially influencing neurotransmitter signaling and synaptic communication. These pathways are essential for maintaining the integrity of the nervous system and are implicated in various neurological and psychiatric conditions. In the context of POD, these signaling pathways may elucidate the mechanisms underlying cognitive decline observed after surgery, particularly in elderly patients. Additionally, enriched pathways such as “Apoptosis,” “Serotonergic synapse,” and “Growth hormone synthesis, secretion, and action” may also influence cognitive function, with reduced growth hormone secretion or action impacting neurogenesis and synaptic function (Sheu et al., 2022).

The results of GO analysis show that the detected miRNAs are significantly enriched in multiple biological processes, molecular functions, and cellular components (Figure 4B). In the “biological process” category, enriched GO terms include “cellular process,” “biological regulation,” and “regulation of biological process” (Figure 4B), suggesting that these miRNAs may be involved in regulating cellular activities and various biological responses. They may play key roles in processes such as neuronal development, apoptosis, and immune response (Chighizola et al., 2019; Faust et al., 2021; Wallace and Pollen, 2024). By modulating intracellular and extracellular signaling pathways and regulatory factors, these miRNAs could affect the function and stability of the nervous system. In the “molecular function” category, enriched GO terms such as “cellular anatomical entity” and “protein-containing complex” indicate that these miRNAs may be involved in maintaining cell structure stability and the assembly and regulation of protein complexes. They could regulate the function of cytoskeletal proteins, membrane proteins, or signaling complexes, participating in intercellular signaling, maintaining cell polarity, and facilitating the formation of neural networks (Thomas, 2017). In the “cellular component” category, enriched GO terms such as “binding” and “catalytic activity” suggest that the genes regulated by these miRNAs may play roles in regulating molecular binding and catalytic reactions, particularly in receptor binding and enzyme activity regulation. By modulating target genes, miRNAs could influence these key molecular processes, potentially affecting physiological processes including neuronal signal transmission and synaptic plasticity, thereby impacting cognitive function in POD context.

## 4 Discussion

Our study identified the miRNA profiles of serum exosomes from clinical patients with POD and those without (non-POD). By comparing miRNA expression levels, we found 16 miRNAs significantly upregulated and 41 miRNAs significantly downregulated in POD patients compared to non-POD patients. We further analyzed the KEGG enrichment pathways of the target genes of these 57 differentially expressed miRNAs, shedding light on the biological functions associated with miRNA dysregulation in POD. Overall, our study aimed to explore miRNA patterns and their functional roles in POD through comprehensive miRNA sequencing and bioinformatics analyses, offering potential insights into clinical biomarkers and therapeutic strategies for POD.

Preoperative prediction and identification of patients at high risk for delirium can minimize the need for frequent screenings and enable timely interventions, which is crucial due to the connection between prolonged delirium and various adverse outcomes. Therefore, a substantial body of research has been dedicated to investigating potential biomarkers for POD. These biomarkers typically encompass indicators obtained from CSF, plasma, and blood (Dunne et al., 2021; Terrando and Akassoglou, 2022). Given the challenges and potential risks associated with CSF collection, there is considerable clinical interest in identifying blood-based biomarkers. Effective biomarkers for POD are those mostly associated with neuroinflammation, disease severity, neurodegenerative processes, and endothelial activation. Inflammatory markers such as IL-6, IL-2, TNF-*α*, and C-reactive protein have been found to be elevated in POD patients compared to those without delirium (Hughes et al., 2016; Nemeth et al., 2017). Additional indicators of neuronal injury, such as S100β, APOE, cortisol, and NSE, have been associated with the onset of delirium post-anesthesia and major surgical procedures (Schaefer et al., 2019). Nonetheless, these biomarkers exhibit limited specificity for predicting the condition and are frequently linked to a range of other pathologies. In contrast, exosomal miRNAs have emerged as potential biomarkers for various diseases due to their stability in bodily fluids like blood, allowing for non-invasive detection. In our study, the 51 significantly different miRNAs identified in serum exosomes from POD patients show potential as preoperative biomarkers. Specifically, miRNAs such as hsa-miR-1299, hsa-miR-320a-3p, hsa-miR-140-3p, hsa-miR-1288-5p, and hsa-miR-210-3p were significantly upregulated in the POD group. And hsa-miR-142-3p was significantly downregulated in the POD group.

Postoperative delirium is characterized by temporary memory impairment and communication difficulties. Our analysis of target genes associated with the significantly detected miRNAs revealed several enriched pathways, with the ‘Neurotrophin signaling pathway’ and the ‘Neuroactive ligand-receptor interaction pathway’ being notably significant. The neurotrophin signaling pathway is critical for neuron survival, growth, and differentiation, and it plays a central role in synaptic plasticity and neuronal function (Han et al., 2021). Disruptions in this pathway have been implicated in various neurodegenerative disorders, including AD and PD (Budni et al., 2015). In the context of POD, impaired neurotrophin signaling could contribute to the cognitive dysfunction observed in elderly patients following surgery (Rahman et al., 2023). It is well-established that aging is associated with a decline in neurotrophic factors, which may exacerbate neuronal vulnerability after stressors such as surgery (Eckenhoff et al., 2020; Khachaturian et al., 2020). Therefore, miRNAs that modulate neurotrophin signaling may play a key role in the pathogenesis of POD. These miRNAs also hold potential as therapeutic targets to preserve cognitive function and prevent POD, particularly in at-risk populations, such as the elderly.

Additionally, the “Neuroactive ligand-receptor interaction pathway” plays a critical role in cognitive function by mediating the actions of neurotransmitters such as dopamine, serotonin, and glutamate (Neff et al., 2021a). These molecules facilitate synaptic transmission, neuronal communication, and brain activity. Altered neurotransmitter receptor activity is a hallmark of several psychiatric and neurodegenerative disorders, including schizophrenia, depression, and epilepsy (Wilson et al., 2023). In the context of POD, disruptions in neurotransmitter signaling may contribute to the acute cognitive decline and delirium observed after surgery. Specifically, impairments in dopamine, serotonin, and glutamate signaling could compromise synaptic plasticity and cognitive processes, thereby exacerbating long-term neurocognitive dysfunction associated with POD. Furthermore, age-related changes in neurotransmitter systems, in combination with surgical stress, may increase the vulnerability to POD by disrupting the balance of these critical signaling pathways.

In addition to these two central pathways, other enriched pathways such as “apoptosis,” “serotonergic synapse,” and “growth hormone synthesis” and action also suggest a broader network of mechanisms that could influence cognitive function in POD. The apoptosis pathway is particularly noteworthy, as cell death in the brain is a potential contributor to the cognitive deficits (Yuan and Yankner, 2000). Dysregulated apoptosis could lead to neuronal loss and affect synaptic connections, impairing brain function (Plascencia-Villa and Perry, 2023a). Furthermore, the “serotonergic synapse” pathway, which is critical for mood regulation and cognition, might be involved in the mood disturbances often seen in POD patients (Pourhamzeh et al., 2022). Reduced growth hormone activity, which plays a role in neurogenesis and synaptic plasticity, has also been linked to cognitive decline and could further exacerbate POD symptoms, especially in the elderly, where growth hormone secretion naturally declines with age (Bartke, 2021; Plascencia-Villa and Perry, 2023a).

Taken together, the miRNAs identified in our study appear to regulate key pathways involved in neuronal survival, synaptic plasticity, and neurotransmitter signaling, all of which are integral to cognitive function. The disruptions in these pathways following surgery could contribute to the onset and progression of POD, particularly in vulnerable populations such as the elderly. Our findings suggest that miRNAs may not only serve as biomarkers for identifying patients at risk for POD but could also represent potential therapeutic targets to mitigate cognitive decline post-surgery. Future studies are needed to further elucidate the precise mechanisms through which these miRNAs influence neurotrophin and neuroactive ligand-receptor pathways and to investigate their potential for use in clinical interventions aimed at preventing or treating POD.

Moreover, inflammation is a key element in the development of POD, as widely reported in numerous studies (Fidalgo et al., 2011; Feng et al., 2017; Yan et al., 2019). In our serum exosomal miRNA analysis of POD patients, KEGG and GO pathway enrichment of target genes revealed associations with the “T17 cell differentiation” and “Immune system process,” both linked to infections and inflammation. Inflammation is widely regarded as a major contributor to POD onset. Again, proving that the miRNAs in serum exosomes in POD holds promise for identifying miRNAs as potential biomarkers.

This study has several limitations that should be acknowledged. Firstly, the sample size for the serum exosomal miRNA microarray analysis was relatively small, which may affect the generalizability of our findings to broader populations. The small sample size might have also constrained our ability to identify additional relevant miRNAs with lower expression levels that could be biologically significant. Future studies with larger cohorts are needed to validate the current findings and further refine the miRNA profiles associated with POD, ensuring that the results are robust and applicable to diverse patient populations. Furthermore, the follow-up duration was limited, as serum was collected only 1 day post-surgery for miRNA sequencing, potentially missing long-term outcomes associated with POD. Different levels and dosages of anesthetics may also influence the circulation of exosomal miRNAs in serum (Kong et al., 2024; Pecorella et al., 2024). Measurement techniques may possess inherent limitations in accuracy and reliability. Lastly, there is a potential for selection bias, as patients were recruited based on specific criteria that may not represent the wider population. These factors warrant careful consideration when interpreting the results of this study.

In summary, we propose that these miRNAs may contribute to the progression of POD, and even to long-term cognitive dysfunction. Serum exosomal miRNAs from POD patients have the potential as prediction biomarkers. Moreover, change of miRNA in serum reflects responses to treatments, providing insights into therapeutic efficacy and disease progression.

## Data Availability

The datasets presented in this study can be found in online repositories. The names of the repository/repositories and accession number(s) can be found in the article/Supplementary material.

## References

[ref1] Androsova G. Krause R. Winterer G. Schneider R. (2015a). Biomarkers of postoperative delirium and cognitive dysfunction. Front. Aging Neurosci. 7:112. doi: 10.3389/fnagi.2015.00112, PMID: 26106326 PMC4460425

[ref2] Bartke A. (2021). Growth hormone and aging. Rev. Endocr. Metab. Disord. 22, 71–80. doi: 10.1007/s11154-020-09593-2, PMID: 33001358

[ref3] Budni J. Bellettini-Santos T. Mina F. Garcez M. L. Zugno A. I. (2015). The involvement of BDNF, NGF and GDNF in aging and Alzheimer’s disease. Aging Dis. 6, 331–341. doi: 10.14336/AD.2015.0825, PMID: 26425388 PMC4567216

[ref4] Chighizola M. Dini T. Lenardi C. Milani P. Podestà A. Schulte C. (2019). Mechanotransduction in neuronal cell development and functioning. Biophys. Rev. 11, 701–720. doi: 10.1007/s12551-019-00587-2, PMID: 31617079 PMC6815321

[ref5] Dobson G. P. (2020). Trauma of major surgery: a global problem that is not going away. Int. J. Surg. 81, 47–54. doi: 10.1016/j.ijsu.2020.07.017, PMID: 32738546 PMC7388795

[ref6] Dunne S. S. Coffey J. C. Konje S. Gasior S. Clancy C. C. Gulati G.. (2021). Biomarkers in delirium: a systematic review. J. Psychosom. Res. 147:110530. doi: 10.1016/j.jpsychores.2021.110530, PMID: 34098376

[ref7] Eckenhoff R. G. Maze M. Xie Z. Culley D. J. Goodlin S. J. Zuo Z.. (2020). Perioperative neurocognitive disorder: state of the preclinical science. Anesthesiology 132, 55–68. doi: 10.1097/ALN.0000000000002956, PMID: 31834869 PMC6913778

[ref8] Faust T. E. Gunner G. Schafer D. P. (2021). Mechanisms governing activity-dependent synaptic pruning in the developing mammalian CNS. Nat. Rev. Neurosci. 22, 657–673. doi: 10.1038/s41583-021-00507-y, PMID: 34545240 PMC8541743

[ref9] Feng X. Valdearcos M. Uchida Y. Lutrin D. Maze M. Koliwad S. K. (2017). Microglia mediate postoperative hippocampal inflammation and cognitive decline in mice. JCI Insight 2:e91229. doi: 10.1172/jci.insight.91229, PMID: 28405620 PMC5374063

[ref10] Fidalgo A. R. Cibelli M. White J. P. M. Nagy I. Maze M. Ma D. (2011). Systemic inflammation enhances surgery-induced cognitive dysfunction in mice. Neurosci. Lett. 498, 63–66. doi: 10.1016/j.neulet.2011.04.063, PMID: 21575676

[ref11] Fiorillo A. Sokhadze E. Sampogna G. (2020). “Neuropsychological and neurophysiological assessment” in Tasman’s psychiatry. ed. Tasman A. (Springer International Press), 1–31.

[ref12] Gao R. Chen C. Zhao Q. Li M. Wang Q. Zhou L.. (2020). Identification of the potential key circular RNAs in elderly patients with postoperative cognitive dysfunction. Front. Aging Neurosci. 12:165. doi: 10.3389/fnagi.2020.00165, PMID: 32655392 PMC7324535

[ref13] Han J. Yoon S. Park H. (2021). Endocytic BDNF secretion regulated by Vamp3 in astrocytes. Sci. Rep. 11:21203. doi: 10.1038/s41598-021-00693-w, PMID: 34707216 PMC8551197

[ref14] Hughes C. G. Pandharipande P. P. Thompson J. L. Chandrasekhar R. Ware L. B. Ely E. W.. (2016). Endothelial activation and blood-brain barrier injury as risk factors for delirium in critically ill patients∗. Crit. Care Med. 44, e809–e817. doi: 10.1097/CCM.0000000000001739, PMID: 27088157 PMC4987204

[ref15] Khachaturian A. S. Hayden K. M. Devlin J. W. Fleisher L. A. Lock S. L. Cunningham C.. (2020). International drive to illuminate delirium: a developing public health blueprint for action. Alzheimers Dement. 16, 711–725. doi: 10.1002/alz.12075, PMID: 32212231

[ref16] Kong X. Lyu W. Lin X. Lin C. Feng H. Xu L.. (2024). Itaconate alleviates anesthesia/surgery-induced cognitive impairment by activating a Nrf2-dependent anti-neuroinflammation and neurogenesis via gut-brain axis. J. Neuroinflammation 21:104. doi: 10.1186/s12974-024-03103-w, PMID: 38649932 PMC11034021

[ref17] Mackowiak S. D. (2011). Identification of novel and known unit 12.10 miRNAs in deep-sequencing data with miRDeep2. Curr. Protoc. Bioinformatics 36, 1–15. doi: 10.1002/0471250953.bi1210s36, PMID: 22161567

[ref18] Martin M. (2011). Cutadapt removes adapter sequences from high-throughput sequencing reads. EMBnet J. 17, 10–12. doi: 10.14806/ej.17.1.200

[ref19] Nation D. A. Sweeney M. D. Montagne A. Sagare A. P. D’Orazio L. M. Pachicano M.. (2019). Blood–brain barrier breakdown is an early biomarker of human cognitive dysfunction. Nat. Med. 25, 270–276. doi: 10.1038/s41591-018-0297-y, PMID: 30643288 PMC6367058

[ref20] Neff R. Kambara K. Bertrand D. (2021a). Ligand gated receptor interactions: a key to the power of neuronal networks. Biochem. Pharmacol. 190:114653. doi: 10.1016/j.bcp.2021.114653, PMID: 34129858

[ref21] Nemeth E. Vig K. Racz K. Koritsanszky K. B. Ronkay K. I. Hamvas F. P.. (2017). Influence of the postoperative inflammatory response on cognitive decline in elderly patients undergoing on-pump cardiac surgery: a controlled, prospective observational study. BMC Anesthesiol. 17:113. doi: 10.1186/s12871-017-0408-1, PMID: 28851286 PMC5576316

[ref22] Ni X. Wu F. Song J. An L. Jiang Q. Bai T.. (2022). Chinese expert consensus on assessment of cognitive impairment in the elderly. Aging Med. 5, 154–166. doi: 10.1002/agm2.12222, PMID: 36247339 PMC9549307

[ref23] Padeiro M. Santana P. Grant M. (2022). “Global aging and health determinants in a changing world” in Aging: From fundamental biology to societal impact. ed. Lavrador M. (Elsevier Press), 3–30.

[ref24] Pecorella G. De Rosa F. Licchelli M. Panese G. Carugno J. T. Morciano A.. (2024). Postoperative cognitive disorders and delirium in gynecologic surgery: which surgery and anesthetic techniques to use to reduce the risk? Int. J. Gynecol. Obstet. 166, 954–968. doi: 10.1002/ijgo.15464, PMID: 38557928

[ref25] Plascencia-Villa G. Perry G. (2023a). Roles of oxidative stress in synaptic dysfunction and neuronal cell death in Alzheimer’s disease. Antioxidants 12:1628. doi: 10.3390/antiox12081628, PMID: 37627623 PMC10451948

[ref26] Pourhamzeh M. Moravej F. G. Arabi M. Shahriari E. Mehrabi S. Ward R.. (2022). The roles of serotonin in neuropsychiatric disorders. Cell. Mol. Neurobiol. 42, 1671–1692. doi: 10.1007/s10571-021-01064-9, PMID: 33651238 PMC11421740

[ref27] Rahman M. M. Islam M. R. Supti F. A. Dhar P. S. Shohag S. Ferdous J.. (2023). Exploring the therapeutic effect of neurotrophins and neuropeptides in neurodegenerative diseases: at a glance. Mol. Neurobiol. 60, 4206–4231. doi: 10.1007/s12035-023-03328-5, PMID: 37052791

[ref28] Reichardt L. F. (2006). Neurotrophin-regulated signalling pathways. Philos. Trans. R. Soc. B Biol. Sci. 361, 1545–1564. doi: 10.1098/rstb.2006.1894, PMID: 16939974 PMC1664664

[ref29] Salemi M. Marchese G. Lanza G. Cosentino F. I. I. Salluzzo M. G. Schillaci F. A.. (2023). Role and dysregulation of miRNA in patients with Parkinson’s disease. Int. J. Mol. Sci. 24:712. doi: 10.3390/ijms24010712, PMID: 36614153 PMC9820759

[ref30] Saliminejad K. Khorram Khorshid H. R. Soleymani Fard S. Ghaffari S. H. (2019). An overview of microRNAs: biology, functions, therapeutics, and analysis methods. J. Cell. Physiol. 234, 5451–5465. doi: 10.1002/jcp.27486, PMID: 30471116

[ref31] Schaefer S. T. Koenigsperger S. Olotu C. Saller T. (2019). Biomarkers and postoperative cognitive function: could it be that easy? Curr. Opin. Anaesthesiol. 32, 92–100. doi: 10.1097/ACO.0000000000000676, PMID: 30507679

[ref32] Sheu S. H. Upadhyayula S. Dupuy V. Pang S. Deng F. Wan J.. (2022). A serotonergic axon-cilium synapse drives nuclear signaling to alter chromatin accessibility. Cell 185, 3390–3407.e18. doi: 10.1016/j.cell.2022.07.026, PMID: 36055200 PMC9789380

[ref33] Silvestro S. Bramanti P. Mazzon E. (2019). Role of miRNAs in alzheimer’s disease and possible fields of application. Int. J. Mol. Sci. 20:3979. doi: 10.3390/ijms20163979, PMID: 31443326 PMC6720959

[ref34] Smoot M. E. Ono K. Ruscheinski J. Wang P. L. Ideker T. (2011). Cytoscape 2.8: new features for data integration and network visualization. Bioinformatics 27, 431–432. doi: 10.1093/bioinformatics/btq675, PMID: 21149340 PMC3031041

[ref35] Swarbrick S. Wragg N. Ghosh S. Stolzing A. (2019). Systematic review of miRNA as biomarkers in Alzheimer’s disease. Mol. Neurobiol. 56, 6156–6167. doi: 10.1007/s12035-019-1500-y, PMID: 30734227 PMC6682547

[ref36] Terrando N. Akassoglou K. (2022). Breaking barriers in postoperative delirium. Br. J. Anaesth. 129, 147–150. doi: 10.1016/j.bja.2022.05.004, PMID: 35718561

[ref37] Theil K. Imami K. Rajewsky N. (2019). Identification of proteins and miRNAs that specifically bind an mRNA in vivo. Nat. Commun. 10:4205. doi: 10.1038/s41467-019-12050-7, PMID: 31527589 PMC6746756

[ref38] Thomas P. D. (2017). The gene ontology and the meaning of biological function. Methods Mol. Biol. 1446, 15–24. doi: 10.1007/978-1-4939-3743-1_2, PMID: 27812932 PMC6438694

[ref39] Wallace J. L. Pollen A. A. (2024). Human neuronal maturation comes of age: cellular mechanisms and species differences. Nat. Rev. Neurosci. 25, 7–29. doi: 10.1038/s41583-023-00760-3, PMID: 37996703

[ref40] Wilson D. M. Cookson M. R. Van Den Bosch L. Zetterberg H. Holtzman D. M. Dewachter I. (2023). Hallmarks of neurodegenerative diseases. Cell 186, 693–714. doi: 10.1016/j.cell.2022.12.032 36803602

[ref41] Yan X. Chan C. Chunling J. Lulong B. (2019). Role of inflammation in perioperative neurocognitive disorders and its therapeutic implication. Chin. Crit. Care Med. 31, 1559–1562. doi: 10.3760/cma.j.issn.2095-4352.2019.12.028 32029052

[ref42] Yuan J. Yankner B. A. (2000). Apoptosis in the nervous system. Nature 407, 802–809. doi: 10.1038/35037739, PMID: 11048732

