# Supplementary material for: Serum-derived exosomal microRNAs as biomarkers for postoperative delirium
Source: Front Neurosci. 2025 Feb 28;19:1525230. doi: 10.3389/fnins.2025.1525230 (PMC11906430; doi:10.3389/fnins.2025.1525230)
Supplement: Supplementary file 1 [file Table_1.docx]

**Supplementary material**

**Serum-derived exosomal microRNAs as biomarkers for postoperative delirium**

Maokai Xu^1†^, Yingjie Chen^1†^, Yujun Lin^2†^, Danfeng Wang^1*^, Xiaochun Zheng^1*^

^1^Department of Anesthesiology, Fujian Provincial Hospital, Fuzhou University Affiliated Provincial Hospital, Fuzhou, China.

^2^Department of Critical Care Medicine, Fuzhou University Affiliated Provincial Hospital, Fuzhou, China

^†^ These authors contributed equally to this work.

Correspondence

Danfeng Wang

251487848@qq.com

Xiaochun Zheng

zhengxiaochun@fjsl.com.cn

**Supplementary tables**

Table 1. RNA Quantification and Quality Assurance by NanoDrop ND-1000

| Sample ID | Sample name | OD260/OD280 ratio | Conc.(ng/μl) | Volme (μl) | Quantity (μg) |
| --- | --- | --- | --- | --- | --- |
| 1 | POD-1 | 1.83 | 1.84 | 22.54 | 10 |
| 2 | POD-2 | 1.85 | 1.82 | 27.32 | 10 |
| 3 | POD-3 | 1.88 | 1.87 | 25.43 | 10 |
| 4 | POD-4 | 1.84 | 1.81 | 23.41 | 10 |
| 5 | POD-5 | 1.86 | 1.83 | 27.87 | 10 |
| 6 | POD-6 | 1.81 | 1.87 | 28.01 | 10 |
| 7 | POD-7 | 1.83 | 1.85 | 30.32 | 10 |
| 8 | Non-POD-1 | 1.89 | 1.82 | 24.82 | 10 |
| 9 | Non-POD-2 | 1.88 | 1.89 | 17.17 | 10 |
| 10 | Non-POD-3 | 1.82 | 1.86 | 19.48 | 10 |

Table 2. miRNAs with Significant Differential Expression in Serum Exosomes of POD Patients Compared to Non-POD Patients

| **miRNA name** | **P-Value** | **Log2FC** | **length** | **seq** |
| --- | --- | --- | --- | --- |
| hsa-miR-320a-3p | 0.00282 | 3.652428514 | 22 | AAAAGCTGGGTTGAGAGGGCGA |
| hsa-miR-1228-5p | 0.003813 | 13.1565874 | 21 | GTGGGCGGGGGCAGGTGTGTG |
| miR-103-z | 0.005649 | -11.03325685 | 25 | AGCAACATTGTACAGGGCTATGAGA |
| hsa-miR-576-3p | 0.005711 | 3.735196287 | 22 | AAGATGTGGAAAAATTGGAATC |
| hsa-miR-6734-5p | 0.007574 | 9.575173262 | 23 | TTGAGGGGAGAATGAGGTGGAGA |
| hsa-miR-641 | 0.010007 | -12.10969574 | 24 | AAAGACATAGGATAGAGTCACCTC |
| hsa-miR-378a-3p | 0.010478 | 2.571628689 | 22 | ACTGGACTTGGAGTCAGAAGGC |
| hsa-miR-142-3p | 0.012543 | -9.767450992 | 23 | TGTAGTGTTTCCTACTTTATGGA |
| hsa-miR-1299 | 0.015021 | 8.144320253 | 22 | TTCTGGAATTCTGTGTGAGGGA |
| miR-27-x | 0.015123 | -12.64173643 | 26 | AGAGCTTAGCTGATTGGTGAACAGTT |
| miR-4508-z | 0.018358 | 12.3232635 | 18 | AAGCGGGGCTGGGCGCGT |
| miR-424-y | 0.018804 | -12.60592547 | 23 | CAAAACGTGAGGCGCTGCTATAA |
| novel-m0019-3p | 0.019414 | -12.4461149 | 21 | TAGGTCTGTGGTTTCTCTAGT |
| miR-148-z | 0.01955 | -12.37814581 | 24 | TCAGTGCATGACAGAACTTGGTTT |
| hsa-miR-6788-5p | 0.019767 | -12.40943867 | 21 | CTGGGAGAAGAGTGGTGAAGA |
| miR-1301-y | 0.021363 | -6.802469101 | 26 | TTGCAGCTGCCTGGGAGTGACTTCTT |
| novel-m0050-5p | 0.021566 | -12.35000451 | 22 | CTGCCTGTCTGTGCCTGCTGTA |
| hsa-miR-4665-5p | 0.021719 | -12.19633266 | 23 | CTGGGGGACGCGTGAGCGCGAGC |
| hsa-miR-6750-5p | 0.025178 | -11.97157948 | 24 | CAGGGAACAGCTGGGTGAGCTGCT |
| hsa-miR-193a-5p | 0.025249 | 2.470564157 | 22 | TGGGTCTTTGCGGGCGAGATGA |
| hsa-miR-205-5p | 0.027214 | -11.74502859 | 22 | TCCTTCATTCCACCGGAGTCTG |
| hsa-miR-7845-5p | 0.027296 | -11.72526826 | 21 | AAGGGACAGGGAGGGTCGTGG |
| novel-m0041-3p | 0.027549 | -11.63284074 | 22 | AGATGCCGGGGTCTCTGTGTGC |
| novel-m0042-3p | 0.027623 | -11.63284074 | 22 | AGATGCCGGGGTCTCTGTGTGC |
| novel-m0044-5p | 0.027779 | -11.61147211 | 22 | AGAGTTGGGAGAGAAGAGAAGA |
| novel-m0043-5p | 0.027933 | -11.62219675 | 24 | AAAATGTGTGGGAAATGAGGAGGA |
| hsa-miR-320b | 0.027942 | 2.168228722 | 22 | AAAAGCTGGGTTGAGAGGGCAA |
| hsa-miR-218-5p | 0.027953 | -11.52268287 | 21 | TTGTGCTTGATCTAACCATGT |
| miR-12136-z | 0.02827 | -11.5111893 | 18 | GAAAAAGTCATGGAGGAA |
| hsa-miR-3164 | 0.029928 | -10.38180614 | 22 | TGTGACTTTAAGGGAAATGGCG |
| hsa-miR-326 | 0.030233 | -11.53655645 | 20 | CCTCTGGGCCCTTCCTCCAG |
| hsa-miR-542-3p | 0.030604 | -11.5582469 | 22 | TGTGACAGATTGATAACTGAAA |
| hsa-miR-4477b | 0.031328 | -11.44638031 | 22 | ATTAAGGACATTTGTGATTGAT |
| novel-m0052-5p | 0.032175 | -11.28692734 | 18 | AGAGCCAGTAGAAACGTG |
| miR-320-y | 0.032261 | 1.368985578 | 24 | AAAAGCTGGGTTGAGAGGGCGAAT |
| miR-5585-y | 0.032484 | -11.24591116 | 19 | GAGTGGCTGGGACTACAGG |
| hsa-miR-423-5p | 0.033223 | 1.65158376 | 23 | TGAGGGGCAGAGAGCGAGACTTT |
| hsa-miR-210-3p | 0.033265 | 9.827264 | 22 | CTGTGCGTGTGACAGCGGCTGA |
| hsa-miR-371a-3p | 0.033684 | -11.28282202 | 23 | AAGTGCCGCCATCTTTTGAGTGT |
| hsa-miR-381-3p | 0.033804 | -11.27007725 | 22 | TATACAAGGGCAAGCTCTCTGT |
| hsa-miR-140-3p | 0.03399 | 1.729879579 | 21 | TACCACAGGGTAGAACCACGG |
| miR-6941-x | 0.03479 | -9.455506155 | 18 | CCCTGGAGGACTGACGTG |
| novel-m0001-3p | 0.035199 | 2.735599765 | 25 | AAAAGCTGGGTTGAGAGGGCGAAAA |
| hsa-miR-374b-5p | 0.036611 | -11.15000321 | 22 | ATATAATACAACCTGCTAAGTG |
| hsa-miR-625-3p | 0.036893 | -11.16843395 | 22 | GACTATAGAACTTTCCCCCTCA |
| hsa-miR-1914-3p | 0.038557 | -10.84937643 | 22 | GGAGGGGTCCCGCACTGGGAGG |
| novel-m0024-5p | 0.038945 | -9.108851117 | 22 | TTGGCGGTGGCTCCTTCTTTGA |
| miR-182-x | 0.039884 | -11.04917241 | 26 | TTTGGCAATGGTAGAACTCACACTGT |
| miR-1843-z | 0.040311 | -8.674288855 | 22 | TATGGAGGTCTCTGTCTGGCTA |
| hsa-miR-532-3p | 0.04131 | -10.81430207 | 22 | CCTCCCACACCCAAGGCTTGCA |
| hsa-miR-6514-5p | 0.043973 | -10.88612586 | 23 | TATGGAGTGGACTTTCAGCTGGC |
| hsa-miR-411-3p | 0.044493 | -10.76418048 | 22 | TATGTAACACGGTCCACTAACC |
| novel-m0067-5p | 0.045822 | -10.4925075 | 19 | AGGAGTGGAACTAGGTTCA |
| hsa-miR-30d-3p | 0.046209 | -10.72112768 | 22 | CTTTCAGTCAGATGTTTGCTGC |
| miR-378-y | 0.046893 | 2.136473478 | 23 | ACTGGACTTGGAGTCAGAAGGAA |
| hsa-miR-329-3p | 0.047442 | -10.7022766 | 22 | AACACACCTGGTTAACCTCTTT |
| novel-m0002-5p | 0.047681 | 8.50439166 | 24 | AGACGGGAGGAAAGAAGGGAGTGG |
